# Supplementary material for: Splice-Junction-Based Mapping of Alternative Isoforms in the Human Proteome
Source: Cell Rep. Author manuscript; Available in PMC 2020 Jan 15. (PMC6961840; doi:10.1016/j.celrep.2019.11.026)

A

sp|Q9Y285|SYFA_HUMAN|ENSG00000179115|RI1|248|chr19|12930527|12930749|−2|r54|T4
PRPPPTQEAHRGLLRRLEASDGGLDSAELAAELGMEHQAVVGAVK q value: 0.0030715 Tr_novel:TRUE RefSeq_Nov
Search result spec prec mz: 1183.6256 Actual spec prec mz: 1183.6255
Fragments matched per AA: 1.29 Proportion of top 20 peaks matched: 0.05

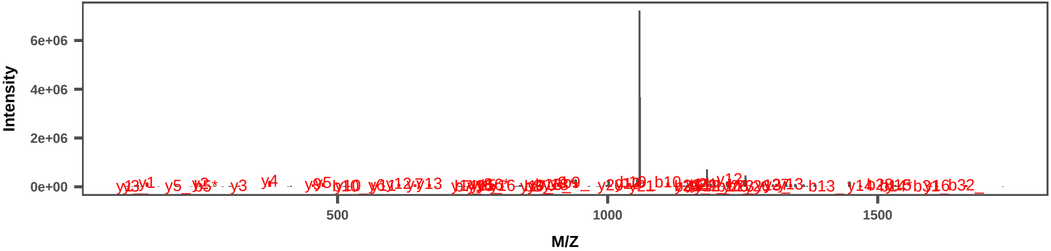

B

Scatterplot of predicted elution time
Fitting R2: 0.742
Novel peptide residual Z score: 2.34
Number of peptides: 3427

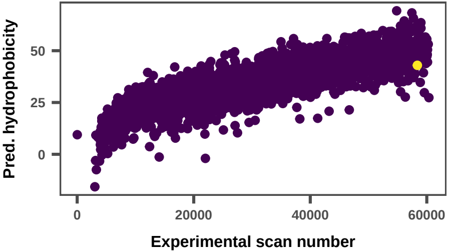

C

Distributions of residuals from best−fit line
of predicted RT vs Expt. scan number
Line: Z score of novel peptide
Z: 2.34

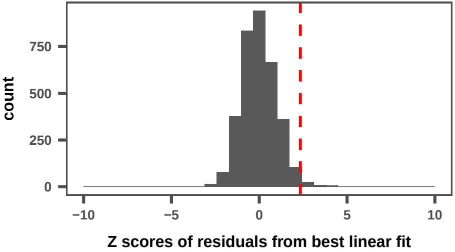

Supplement: 2 [file NIHMS1546469-supplement-2.zip › DF1/PXD009737/TestisLysC/TestisLysC_28_FARSA_PRPPPTQEAHRGLLRRLEASDGGLDSAELAAELGMEHQAVVGAVK.pdf]
